# Commentary on Special Issue “Fetal Growth: What Is New in the Clinical Research?”

**DOI:** 10.3390/jcm11195795

**Published:** 2022-09-29

**Authors:** Erich Cosmi, Silvia Visentin

**Affiliations:** Department of Woman’s and Child’s Health, Padova University, 35100 Padua, Italy

Fetal growth restriction (FGR) is a common complication of pregnancy (3–10%) and has been associated with a variety of adverse perinatal outcomes. It represents a leading cause of perinatal mortality, most common at 28–31 weeks of gestation, and short-term morbidity, such as perinatal asphyxia, meconium aspiration, hypothermia, hypoglycemia, polycythemia, jaundice, feeding difficulties, necrotizing enterocolitis, and late-onset sepsis [1,2,3]. 

Furthermore, changes in the fetal nutritional environment, prenatal programming, and postnatal catch-up growth in FGR infants lead to long-term adverse consequences such as neurodevelopmental impairment, increased risk of cardiovascular disease, and metabolic syndrome that span over a lifetime. Therefore, their identification becomes important for neonatal and long-term pediatric management (Barker’s theory) [4,5].

Currently, FGR continues to be a challenging problem for clinicians despite advances in both obstetric and neonatal care.

The incidence of FGR varies among populations and increases with decreasing gestational age. In addition, it is challenging to interpret the literature as some studies use the term small for gestational age (SGA), which may include both infants who are FGR as well as those who are constitutionally normally small. [6].

Approximately 10% of term infants in developed countries are SGA, compared with 20 percent of term infants in resource-limited countries. Data from large cohorts (CHERG, 2012) showed that 22 percent of neonatal deaths occurred in infants born SGA [7].

About the etiology, even if FGR can result from multiple causes, such as genetic (one-third of infants), epigenetic, environmental, hormonal regulation, or vascular troubles and their potential interactions, in approximately 60% of FGR cases, they are idiopathic and multifactorial [8].

Additionally, the reference birth weight and intrauterine growth charts will influence the classification and incidence of FGR. In particular, ultrasound assessments of estimated fetal weight across gestation or/and birthweights of preterm, term, and post-term newborns are the most frequent curves used [9].

Moreover, several population-based studies have developed specific fetal growth nomograms for specific populations. Comparing intrauterine and birthweight-derived standards, it emerged that neither accurately predicted neonatal morbidity and mortality, but intrauterine curves performed quite better than the second ones [10,11,12]. In this contest, customized growth curves have been developed, improving the accuracy of assessment of fetal size and growth, considering maternal factors such as parity, height, and weight, and by fetal sex [13]. However, conflicting data are present in the literature about this aspect. Finally, screening of the low risk population to detect FGR or SGE fetuses by ultrasound is not recommended as it has not been proven to decrease perinatal mortality. If a single examination is performed, it should be carried out close to 36 weeks as better detects SGA and/or FGR fetuses than a sonogram at 32 weeks. If two screening examinations are to be performed, then it is reasonable to time them for 32 and 36 weeks [14].

So, what has been said so far reveals how there is a lack of consensus regarding terminology, etiology, and diagnostic criteria for FGR, with uncertainty surrounding the optimal management and timing of delivery.

This Special Issue intended to investigate the state of the art regarding the FGR topic, in particular, new points of view, regarding the pathophysiology, diagnosis, and treatment. Two papers studied the predictivity of pre-birth ultrasound control, at different gestational weeks and using different reference curves, about the definition of FGR at delivery. One assessed neonatal outcome based on whether the condition of FGR in utero was known. Finally, two papers investigated the mechanisms of glucose transport, the primary fetal energy source, in fetuses with different FGR classifications, and the identification of which miRNAs were expressed in FGR fetuses of smoking mothers.

*Ricardo Savirón-Cornudella* et al. retrospectively investigated 9585 singleton pregnancies, with the aim to compare the ability of ultrasound-estimated percentile weight—according to six growth standards, by ultrasound at 35 weeks, including population, population-customized, and international references—to predict late-onset SGA, defined as a birth weight below the 10th percentile at term delivery. The six curves were the customized Miguel Servet University Hospital (MSUH), Figueras standards, the non-customized MSUH, the Fetal Medicine Foundation (FMF), the INTERGROWTH-21st, and WHO standards. They obtained that, for a 10% FPR, the detection rates for SGA for all standards ranged between 48.9% with the Figueras standard (AUC: 0.82) and 60.8% with the Fetal Medicine Foundation standard (AUC: 0.87). The Fetal Medicine Foundation and the non-customized MSUH standards showed greater SGA prediction ability than the Intergrowth-21st, Figueras, and WHO standards. In conclusion, there is a significant advantage in the non-customized MSUH and Fetal Medicine Foundation standards and in the adverse perinatal outcome (APO) detection rate.

An ultrasound at 35 weeks (range 34+0–36+6) is a predictor of SGA fetuses at delivery at term. The detection rate of SGA at delivery by ultrasound between 33 and 34 weeks is approximately 52%, while between 36 and 37 weeks, it is approximately 60%. It is not possible to delay a ultrasound universally to 37 weeks because early FGRs would not be detected.

Moreover, the authors recommended raising the ultrasound-estimated weight percentile cutoff point above 10 for fetal growth control, at least from the 10th to the 20th percentile, between 35 and 36 weeks because the 10th percentile presented a low predictive capacity for SGA at delivery.

Finally, considering the second aim, the authors showed higher detection rates as intervals decreased (1–6 weeks).

*María Sonsoles Galán Arévalo* et al. aimed to evaluated whether newborns appropriate for gestational age (AGA) who have suffered a reduction in estimated fetal growth by a decrease of 20 or more centiles between the 35th gestational week and delivery had similar APOs to those diagnosed with SGA. The fact that most adverse perinatal outcomes occurred in AGAs has postulated that some newborns with a weight percentile between 10 and 90 also did not reach their growth potential and could be considered FGR.

This was an observational, retrospective cohort study and included 1067 pregnant women. It is part of the great Growth Declining Newborns (GROWIN) study conducted at the Villalba University General Hospital, Spain. Considering that the stillbirth risk increases up to eight-fold in the presence of undetected FGR, prenatally identification becomes essential and several preventive strategies are focused on precise prenatal detection. The highest rate and risk of APOs were in fact SGAs, and the only group that presented a significant risk corresponded to the SGA non-FGD group. However, there was no differences between AGA detected and non-detected in terms of APOs; if the decrease in percentile cutoffs is over 40, the risk of cesarean section due to non-reassuring fetal status rises. In any case, the risk of APO increases if the birthweight percentile is less than 10.

Regarding this topic, Chiara Lubrano et al. have considered the impact of different restricted fetuses on APOs. This study was retrospective and analyzed the data collected in the maternal–fetal medicine center of the Buzzi Hospital in Milan. The fetuses were distinguished in AGA, SGA, and FGR, early and late. Maternal characteristics, mode of conception, weight gain during pregnancy, and the onset of any obstetric pathologies were analyzed in all groups. Additionally, timing and mode of delivery, infant and placental weights, Apgar scores at 1 and 5 min after birth, and umbilical arterial blood gas and acid–base values (pH, base excess, and lactate as a blood gas analyzer) have been collected and recorded as the short-term infant outcomes. The authors concluded that age (higher in early FGR), being primiparous (higher frequency in FGR and SGA), pregestational weight and BMI (lower in both FGR and SGA), and gestational weight gain (reduced in early FGR), are important risk factors for a reduced fetal potential growth. About SGA, they showed higher lactates and lower base excess values compared to the other groups; unexpected ones born by vaginal delivery, managed as AGA, were more hyperlactacidemic and hypoxemic. However, neonatal outcomes (accesses and days of hospitalization in the NICU) were better than FGR, likely due to gestational age and birthweight similar to AGA. Nevertheless, these fetuses showed pH values in the normal range, suggesting an optimal delivery timing and mode. The authors concluded that an early identification, together with an optimization of delivery times, reduces the fetal morbidity and mortality of the fetuses themselves. In particular, they underlined the worsening stress adaptation of labor for SGA infants, resulting in hyperlactacidemic and hypoxemic states at birth. The fetal growth, either restricted or increased, depends on the condition of abnormal utero-placental vascularity; hypoxia; and reduced or exaggerated transplacental maternal availability of nutrients, in particular glucose. Glucose is the primary energy substrate utilized by the fetus; its crosses the placenta by facilitated diffusion, and it remains closely dependent on the maternal serum concentration. GLUT family members are responsible for its transport and different substrate specificity, kinetics, and localization.

*Paweł Jan Stanirowski* et al. studied the expression of glucose transporter proteins GLUT-1, GLUT-3, GLUT-8, and GLUT-12 in the placenta of macrosomic (n 26), SGA (n 11), FGR (13), and control fetuses (n 20). A total of 70 placental tissue samples were analyzed through the computer-assisted quantitative morphometry and vascular density evaluation. The conclusion was that idiopathic fetal macrosomia was not associated with changes in the placental expression of the GLUT proteins studied. On the contrary, SGA placentas presented an expression of GLUT-8 significantly decreased respect the other groups. The significance of this result remains unclear; animal models observed an abnormal process of decidualization, potentially leading to impaired placentation and aberrant fetal growth. Finally, FGR placenta showed a significant decrease in GLUT-1 and increase in GLUT-3 protein expression. The decrease in GLUT-1 observed could be secondary to reduced oxygen delivery to the placenta and a compromised vascularization. This hypoxic condition triggers a compensatory mechanism, which is expressed by an increase in the expression of GLUT 3, which presents higher affinity to glucose than GLUT-1. The most obvious reason for the observed differences in GLUT expression between the FGR and SGA groups is the more severe placental pathology present in the former.

The condition of placenta hypoxia has been extensively studied in FGR placenta.

Tobacco negatively affects fetal growth, in particular length, weight, and head circumference. Not all of the studies, also those in animals, are completely consistent; if some reported a significant reduction in fetal growth curve, others documented direct tissue damage and impaired placental circulation. Epigenomic studies have shown the association between tobacco and DNA methylation in cord blood and newborns, some reversible postnatally.

*Eva Barrio* et al., in their article, aimed to study the impact of smoking during pregnancy and its role in intrauterine growth restriction via hypermethylated miRNAs. Tobacco may be associated with an increase in miRNA genes methylation and, therefore, altered miRNA expression in newborns until adolescence. This could have an effect on the correct development of the placenta and uteroplacental arteries, directly or indirectly, causing vasoconstriction and increasing uterine vascular resistance. The final is a reduced fetal growth.

This was a transversal study conducted in newborns in Zaragoza, Spain. The inclusion criteria were low-birth-weight newborns exposed to tobacco smoke (more than 10 cigarettes per day during the first trimester of pregnancy) and normal-weight newborns not exposed to tobacco smoke. They were selected consecutively until obtaining 10 patients (5 smoking and 5 not). A cord blood sample was obtained during delivery. Seven hypermethylated miRNAs were identified in the cord blood of low-birth-weight newborns of smoking mothers. They are involved in fetal development by regulating processes such as placental angiogenesis, fetal–placental growth, and fetal oxygenation mechanisms. The authors correlated the results with the fetal programming hypothesis, which postulates that a hostile intrauterine environment and, therefore, a restriction of growth as an adaptation mechanism can determine metabolic and cardiovascular changes in both pediatric and adult age. The main limitation is in the lack of functional studies relating a specific miRNA to an associated pathology.

Further studies are needed to better define this obstetric pathology that has not yet found a complete explanation and an optimal treatment to avoid unexpected adverse events.
